# The Effect of Physical Activity on Glycemic Variability in Patients With Diabetes: A Systematic Review and Meta-Analysis of Randomized Controlled Trials

**DOI:** 10.3389/fendo.2021.767152

**Published:** 2021-11-17

**Authors:** Xingyun Zhu, Lina Zhao, Jing Chen, Chu Lin, Fang Lv, Suiyuan Hu, Xiaoling Cai, Li Zhang, Linong Ji

**Affiliations:** ^1^ Department of Endocrinology and Metabolism, Peking University People’s Hospital, Beijing, China; ^2^ Department of Endocrinology and Metabolism, Langfang Traditional Chinese Medicine (TCM) Hospital, Hebei, China; ^3^ China Institute of Sport Science, Beijing, China

**Keywords:** physical activity, glycemic variability, glycemic control, diabetes, continuous glucose monitoring

## Abstract

**Purpose:**

The effect of physical activity on glycemic variability remains controversial. This meta-analysis aimed to assess the overall effect of physical activity treatment on glycemic variability in patients with diabetes.

**Methods:**

PubMed/MEDLINE, Embase, and Cochrane databases were searched for clinical trials that conducted in patients with type 1 diabetes mellitus and type 2 diabetes mellitus with reports of the mean amplitude of glycemic excursion (MAGE), time in range (TIR), time above range (TAR), or time below range (TBR). Eligible trials were analyzed by fixed-effect model, random effect model, and meta-regression analysis accordingly.

**Results:**

In total, thirteen trials were included. Compared with the control group, physical activity intervention was significantly associated with increased TIR (WMDs, 4.17%; 95% CI, 1.11 to 7.23%, P<0.01), decreased MAGE (WMDs, -0.68 mmol/L; 95% CI, -1.01 to -0.36 mmol/L, P<0.01) and decreased TAR (WMDs, -3.54%; 95% CI, -5.21 to -1.88%, P<0.01) in patients with diabetes, but showed insignificant effects on TBR. Patients with higher baseline BMI levels was associated with a greater decrease in MAGE (β=-0.392, 95% CI: -0.710, -0.074), and patients with lower baseline HbA1c levels was associated with a greater increase in TBR during physical activities (β=-0.903, 95% CI: -1.550, -0.255).

**Conclusion:**

Physical activity was associated with significantly decreased glycemic variability in patients with diabetes. Patients with higher BMI might benefit more from physical activity therapy in terms of a lower MAGE. Hypoglycemia associated with physical activity treatment still warranted caution, especially in patients with intensive glycemic control.

**Systematic Review Registration:**

PROSPERO [CRD42021259807].

## Introduction

Physical activity was considered as an easy and economical way to improve overall well-being (1, 2). In previous studies, physical activity was proved to improve glycemic control, manifested as reducing hemoglobin A1c (HbA1c) and fasting plasma glucose (FPG) (3–6). Recent studies indicated that even light physical activities breaking the prolonged sitting could bring metabolic benefits to patients with diabetes (7, 8). Hence, physical activity was recognized as a key non-pharmaceutical strategy for patients with diabetes in most international and national guidelines (9–11).

However, the efficacy of physical activity treatment on the improvement of HbA1c could only indicate the reduction in mean glucose level. Whether taking physical activities will expose diabetic patients to an increased risk of hyperglycemia or hypoglycemia is still under great debate (4, 5, 12–17). Since emerging evidence has built a positive relationship between glycemic variability and diabetes complications, the effect of physical activity on glycemic variability increasingly attracts public attention (18–21).

Accessing glycemic variability was not easy with traditional glucose monitor strategies, including self-monitoring of blood glucose, HbA1c measurement, and glycated albumin measurement. Nevertheless, with the availability of new glucose monitoring technics, especially continuous glucose monitoring (CGM), several computations turned out to be good indicators for glycemic variability. Among those computations, time in range (TIR), time above range (TAR), time below range (TBR), and mean amplitude of glycemic excursion (MAGE) were the most popular indicators for within-day glycemic variability and were wildly discussed in previous researches (22–24).

In this study, we used TIR, TAR, TBR, and MAGE to measure the glycemic variability. The main purpose of this meta-analysis was to evaluate the effect of physical activity treatment on glycemic variability in patients with diabetes.

## Materials and Methods

This meta-analysis was conducted according to the Cochrane Handbook for Systematic Reviews of Interventions (25). A PRISMA (Preferred Reporting Items for Systematic Reviews and Meta-Analyses) checklist was created and showed in the **Supplementary Materials**. The study was previously registered on PROSPERO with ID CRD42021259807.

### Data Sources and Searches

According to the Cochrane Handbook (25), one investigator (Xingyun Zhu) performed the systematic searches in EMBASE, MEDLINE (accessed by PubMed), and Cochrane Central Register of Controlled Trials for studies of physical activity intervention published between January 1980 and October 2021. The searching terms were as follows: “Exercise”, “Physical Activity”, “Circuit-Based Exercise”, “Cool-Down Exercise”, “Warm-Up Exercise”, “Exercise Movement Techniques”, “Exercise Therapy”, “Exercise Test”, “Resistance Training”, “Muscle Stretching Exercises”, “High-Intensity Interval Training”, “Diabetes Mellitus”, “Diabetes”, “Diabetes Mellitus”, “Randomized Controlled Trial”, “Randomized Controlled Trials”, “Controlled Clinical Trial”, “Continuous Glucose Monitoring”. The full electronic search strategy was shown in **Supplementary Table S1**.

### Study Selection

The inclusion criteria for the meta-analysis were as follows: 1) Random controlled trials (RCTs) conducted in people with type 1 diabetes or type 2 diabetes. 2) RCTs that had at least one arm of physical activity intervention, compared with a no-exercise control group. 3) RCTs that monitored glucose variation with CGM. 4) RCTs with the reported result of any of the following as outcome variables: MAGE, TIR, TAR, TBR. The exclusion criteria for the meta-analysis were as follows: 1) RCTs conducted in patients with gestational diabetes, other special types of diabetes, or without diabetes. 2) Trials conducted in animal models. 3) Duplicate publications. 4) Reviews. In this study, physical activity was defined as any intervention that breaks prolong sitting.

### Data Extraction and Quality Assessment

Two investigators (Xingyun Zhu and Lina Zhao) independently collected data of all studies, including publication details, population characteristics, physical activities, results of TIR, TAR, TBR, MAGE, etc. Definitions of hyperglycemia and hypoglycemia were also recorded. We invited a third investigator (Chu Lin) to join the discussion and resolved discrepancies by consensus. The risk of bias was evaluated according to the Cochrane risk of bias tool. The quality of the included evidence was evaluated by the GRADE Approach (26).

### Data Synthesis and Analysis

The primary outcome was the changes in TIR and MAGE between the physical activity treatment group and the control group in patients with diabetes. The secondary outcomes included the changes in TAR and TBR between the physical activity treatment group and the control group in patients with diabetes.

Prespecified subgroup analyses were stratified by baseline age, sex, BMI, HbA1c, and disease duration. All the outcomes in analyses were continuous variables and evaluated by computing the weighted mean differences (WMDs) and 95% confidence intervals (CI). We used Higgins I^2^ statistics to evaluate between-study heterogeneity, with an I^2^ value >50 indicating a high level of heterogeneity. The prediction intervals of I2 were calculated according to a method described by previous studies (27, 28). The fixed-effects model was used for a low level of heterogeneity, and the random-effects model was used for a high level of heterogeneity. We used the Z test to compare the mean difference of effect sizes between subgroups. Meta-regression analyses were conducted to evaluate the associations between different potential influencing indicators (including age, sex, disease duration, baseline HbA1c level, body mass index) in outcomes. Publication bias was assessed *via* the Beggs test and funnel plot. Statistical significance was considered at *P *< 0.05.

## Results

### Study Selection, Study Characteristics, and Quality Assessment

**Figure 1:**
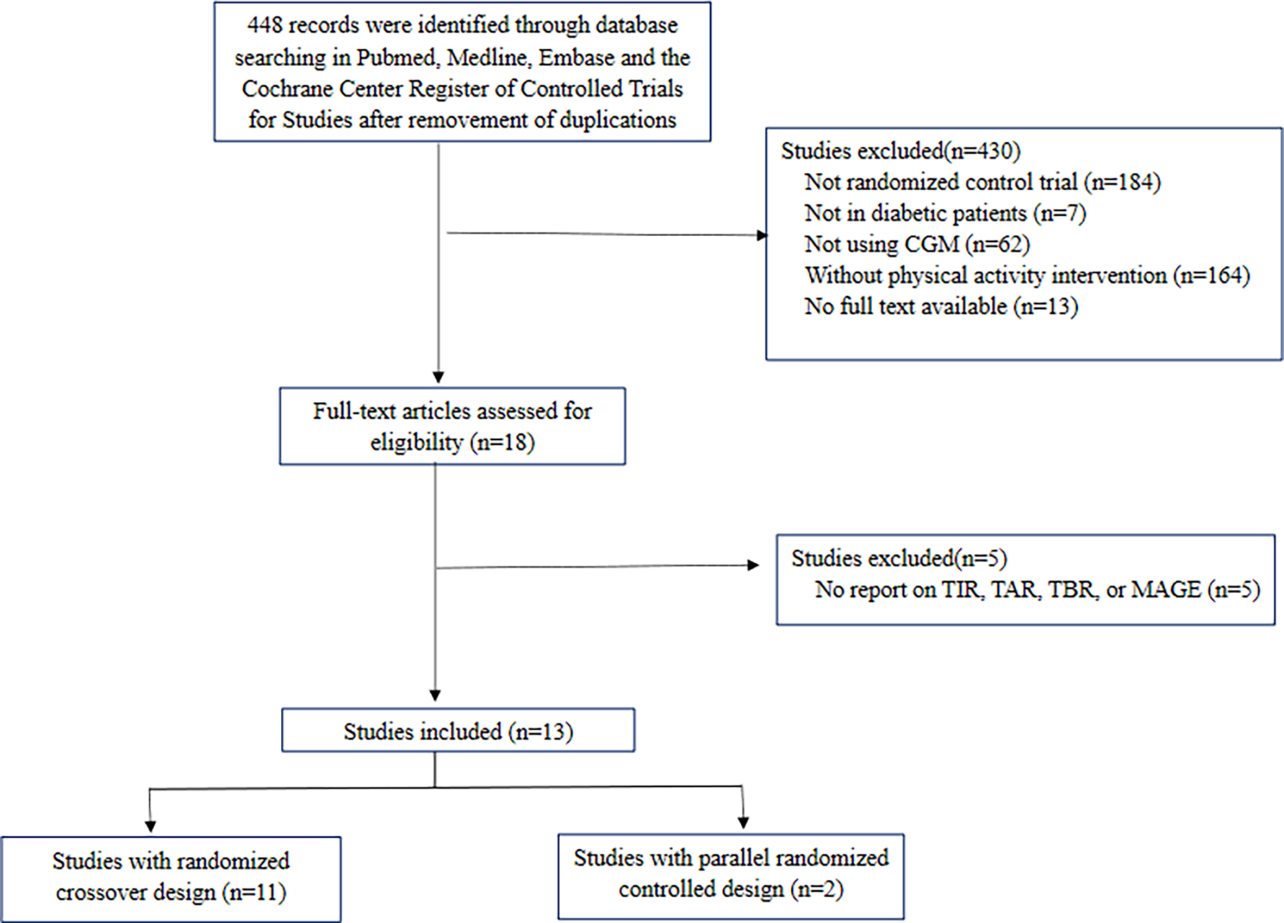
Flow chart of included trials. CGM, continuous glucose monitoring; TIR, time in range; TAR, time above range; TBR, time below range; MAGE, mean amplitude of glycemic excursion.

In total, 13 RCTs were included in this meta-analysis. Among all included trials, 11 were studies with crossover design, and 2 were studies with parallel design. **Figure 1** summarized the selection process of included studies. Baseline characteristics of each included RCT were shown in **Table 1**. The descriptions of exercise in RCTs were summarized in the supplementary material (**Supplementary Table S2**). The overall risk of bias and selective reporting bias was low, and no significant publication bias was found (**Supplementary Table S3** and **Supplementary Figures S1**, **S2**). According to the GRADE Approach, the quality of included trials in included outcomes was ranked low to moderate (**Supplementary Table S4**).

**Table 1:** Baseline characteristics of each included random controlled trials.

Author, year	Exercise duration	Treatment group	Standard diet	No. of patients	Age (years)	Men (%)	BMI (kg/m^2^)	Duration of diabetes (years)	Baseline HbA1c (%)	Definition of hypoglycemia and hyperglycemia (mmol/L)
Paddy 2017 (29)	24 hours	Light-intensity walking	yes	24	62 ± 6	58.3	33.0 ± 3.4	6.8 ± 5.1	7.2 ± 0.7	3.9≤; 10≥;
		Simple resistance activities		24	62 ± 6	58.3	33.0 ± 3.4	6.8 ± 5.1	7.2 ± 0.7	
		Control		24	62 ± 6	58.3	33.0 ± 3.4	6.8 ± 5.1	7.2 ± 0.7	
Jonida 2015 (30)	72hours	Continuous walking	yes	9	58.2 ± 6.6	100	30.2 ± 3.1	5.2 ± 4.3	7.0 ± 0.6	NA; NA;
		Split walking		9	58.2 ± 6.6	100	30.2 ± 3.1	5.2 ± 4.3	7.0 ± 0.6	
		Control		9	58.2 ± 6.6	100	30.2 ± 3.1	5.2 ± 4.3	7.0 ± 0.6	
Jordan 2019 (31)	6 days	Exercise	yes	63	64.4 ± 8.0	46	30.5 ± 6.5	9.7 ± 6.1	6.8 ± 0.7	4≤; 10≥;
		Control		63	64.4 ± 8.0	46	30.5 ± 6.5	9.7 ± 6.1	6.8 ± 0.7	
Zheng Li 2018 (32)	2days	Exercise	yes	29	51 ± 11.2	75.9	24.8 ± 3.4	5.7 ± 6.0	7.3 ± 1.3	NA; NA;
		Control		29	51 ± 11.2	75.9	24.8 ± 3.4	5.7 ± 6.0	7.3 ± 1.3	
Richard 2018 (33)	3days	Reduced-exertion high-intensity interval training (REHIT)	yes	11	52 ± 6	100	29.7 ± 3.1	4 ± 3	7.0 ± 0.8	NA; 9≥;
		Moderate–vigorous-intensity continuous exercise 30 min (MICT)		11	52 ± 6	100	29.7 ± 3.1	4 ± 3	7.0 ± 0.8	
		High-intensity interval training (HIIT)		11	52 ± 6	100	29.7 ± 3.1	4 ± 3	7.0 ± 0.8	
		Control		11	52 ± 6	100	29.7 ± 3.1	4 ± 3	7.0 ± 0.8	
Kristian 2016 (34)	2 weeks	Continuous walking training	NA	14	65 ± 2	78.6	NA	NA	6.6 ± 0.4	4≤; 10≥;
		Interval walking training		14	65 ± 2	78.6	NA	NA	6.5 ± 0.3	
		Control		14	65 ± 2	78.6	NA	NA	6.6 ± 0.3	
Myette-Côté 2015 (35)	24 hours	Exercise	yes	10	59.0 ± 9.6	50	29.5 ± 4.7	7.7 ± 5.2	NA	4≤; 8≥;
		Control		10	59.0 ± 9.6	50	29.5 ± 4.7	7.7 ± 5.2	NA	
Tasuku 2016 (36)	24 hours	Moderate-intensity continuous exercise	yes	10	60 ± 6	80.0	30.8 ± 5.4	6.8 ± 4.6	7.1 ± 1.0	4≤; 10≥;
		High-intensity interval exercise		10	60 ± 6	80.0	30.8 ± 5.4	6.8 ± 4.6	7.1 ± 1.0	
		Control		10	60 ± 6	80.0	30.8 ± 5.4	6.8 ± 4.6	7.1 ± 1.0	
Jennifer 2018 (37)	24hours	Morning walk after breakfast	yes	30	64 ± 8.2	46.7	31.7 ± 5.4	10 ± 7.8	7.4 ± 1.1	NA; 10≥;
		Post-meal breaks from sitting		30	64 ± 8.2	46.7	31.7 ± 5.4	10 ± 7.8	7.4 ± 1.1	
		Control		30	64 ± 8.2	46.7	31.7 ± 5.4	10 ± 7.8	7.4 ± 1.1	
Matthew 2020 (38)	12 days	Morning Exercise	yes	14	65 ± 9.0	57.1	27.2 ± 3.5	10.5 ± 6.7	6.7 ± 0.6	4≤; 10≥;
		Afternoon Exercise		14	65 ± 9.0	57.1	27.2 ± 3.5	10.5 ± 6.7	6.7 ± 0.6	
		Evening Exercise		14	65 ± 9.0	57.1	27.2 ± 3.5	10.5 ± 6.7	6.7 ± 0.6	
		Control		14	65 ± 9.0	57.1	27.2 ± 3.5	10.5 ± 6.7	6.7 ± 0.6	
Ravi 2018 (39)	3weeks	Aerobic exercise	yes	10	33 ± 6	40.0	24.4 ± 2.1	18 ± 10	7.4 ± 1	3.9≤; 10≥;
		Resistance exercise		10	33 ± 6	40.0	24.4 ± 2.1	18 ± 10	7.4 ± 1	
		Control		10	33 ± 6	40.0	24.4 ± 2.1	18 ± 10	7.4 ± 1	
Kamilla 2018 (40)	11 weeks	Endurance training	yes	12	58 ± 8	58.3	27.4 ± 3.1	6 ± 4	6.9 ± 0.9	3.9≤; 10≥;
		High-intensity interval training		13	54 ± 6	53.8	28.1 ± 3.5	8 ± 4	6.8 ± 0.8	
		Control		7	57 ± 7	71.4	28.0 ± 3.5	7 ± 5	7.0 ± 1.15	
Angela 2020 (41)	12 weeks	High-intensity interval training	NA	12	40.5 ± 10.0	50	29.0 ± 2.1	15.8 ± 12.2	8.63 ± 0.66	3.9≤; 10≥;
		Control		15	46.1 ± 10.5	67	31.6 ± 3.4	22.5 ± 10.0	8.37 ± 0.71	

HbA1c, Hemoglobin A1c; BMI, body mass index; NA, not available.

### The Effect of Physical Activity on the TIR

Compared with the control group, patients that took physical activity had significantly higher TIR (WMDs, 4.17%; 95% CI, 1.11 to 7.23%, *P*<0.01, I^2^ = 0%). In patients with type 2 diabetes, compared with the control group, TIR was significantly increased in patients with physical activity treatment (WMDs, 4.21%; 95% CI, 0.95 to 7.46%, *P*<0.01, I^2^ = 0%). However, in patients with type 1 diabetes, TIR was not significantly different between groups (**Table 2** and **Supplementary Figure S3**).

**Table 2:** Effect of physical activity on TIR, MAGE, TAR, and TBR.

Group or Subgroup	No. of participants (physical activity/control)	WMDs	95% CI of WMDs	P value	I^2^	95% CI of I^2^
Effect of physical activity on TIR						
Overall	120/112	4.17%	1.11%, 7.23%	<0.01	0%	0%, 75%
T1DM	32/35	3.87%	-5.04%, 12.78%	0.39	1%	0%, 90%
T2DM	88/77	4.21%	0.95%, 7.46%	<0.01	0%	0%, 90%
Effect of physical activity on MAGE						
Overall	303/311	-0.68mmol/L	-1.01 mmol/L, -0.36 mmol/L	<0.01	78%	66%, 86%
HbA1c <7%	143/143	-0.38mmol/L	-0.81 mmol/L, -0.04 mmol/L	0.08	60%	9%, 83%
HbA1c ≥7%	160/168	-1.13mmol/L	-1.44 mmol/L, -0.82 mmol/L	<0.01	52%	7%, 75%
BMI≥30kg/m^2^	161/169	-1.08mmol/L	-1.45 mmol/L, -0.71 mmol/L	<0.01	68%	37%, 84%
BMI<30kg/m^2^	114/114	-0.44mmol/L	-0.82 mmol/L, -0.06 mmol/L	0.02	0%	0%, 68%
Age ≤ 60 years	122/130	-0.50mmol/L	-0.93 mmol/L, -0.06 mmol/L	0.03	0%	0%, 66%
Age>60 years	181/181	-0.77mmol/L	-1.18 mmol/L, -0.36 mmol/L	<0.01	89%	81%, 94%
Male percentage ≤ 75%	192/192	-0.80mmol/L	-1.19 mmol/L, -0.42 mmol/L	<0.01	82%	66%, 91%
Male percentage>75%	111/119	-0.58mmol/L	-1.02 mmol/L, -0.14 mmol/L	<0.01	40%	0%, 71%
Disease duration≥7.1 years	143/143	-0.38mmol/L	-0.81 mmol/L, 0.04 mmol/L	0.08	60%	9%, 83%
Disease duration<7.1 years	160/168	-1.13mmol/L	-1.44 mmol/L, -0.82 mmol/L	<0.01	58%	52%, 90%
Effect of physical activity on TAR						
Overall	265/265	-3.54%	-5.12%, -1.88%	<0.01	0%	0%, 49%
T1DM	32/35	-1.43%	-11.07%, 8.30%	0.77	40%	0%, 81%
T2DM	233/230	-3.61%	-5.30%, -1.92%	<0.01	0%	0%, 52%
Effect of physical activity on TBR						
Overall	95/87	0.36%	-1.79%, 2.51%	0.74	92%	87%, 95%
T1DM	32/35	-0.89%	-5.22%, 3.44%	0.69	34%	0%, 78%
T2DM	63/52	0.69%	-1.78%, 3.15%	0.58	95%	92%, 97%

HbA1c, Hemoglobin A1c; BMI, body mass index; T1DM, type 1 diabetes; T2DM, type 2 diabetes; TIR, time in range; TAR, time above range; TBR, time below range; MAGE, mean amplitude of glycemic excursion; WMDs, weighted mean differences; CI, confidential interval.

### The Effect of Physical Activity on the MAGE

Overall, compared with the control group, physical activity led to significant reductions in MAGE in patients with type 2 diabetes (WMDs, -0.68 mmol/L; 95% CI, -1.01 to -0.36 mmol/L, *P*<0.01, I^2^ = 78%) (**Table 2** and **Supplementary Figure S4**).

Compared with the control group, MAGE was significantly decreased in patients with baseline HbA1c ≥7% in the physical activity group (WMDs, -1.13 mmol/L; 95% CI, -1.44 to -0.82, *P*<0.01, I^2^ = 52%), but not in patients with baseline HbA1c<7% in the physical activity group. When stratified by baseline BMI level, compared with the control group, MAGE was significantly decreased in patients with BMI≥30 kg/m^2^ in the physical activity group (WMDs, -1.08 mmol/L; 95% CI, -1.45 to -0.71, *P*<0.01, I^2^ = 68%), as well as in patients with BMI<30 kg/m^2^ in physical activity group (WMDs, -0.44 mmol/L; 95% CI, -0.82 to -0.06, *P*<0.01, I^2^ = 0%). When compared with the control group, a significant decrease in MAGE was revealed in patients with shorter disease duration in the physical activity group (WMDs, -1.13 mmol/L; 95% CI, -1.44 to -0.82, *P*<0.01, I^2^ = 58%), but was not observed in patients with longer disease duration in the physical activity group (**Table 2**).

### The Effect of Physical Activity on the TAR and TBR

Overall, physical activity was associated with significant decrease in TAR (WMDs, -3.54%; 95% CI, -5.21 to -1.88%, *P*<0.01, I^2^ = 0%) with no effect on TBR (WMDs, 0.36%; 95% CI, -1.79 to 2.51%, *P*=0.74, I^2^ = 92%) when compared to the control group.

When compared to the control group, physical activity was associated with a significant decrease in TAR (WMDs, -3.61%; 95% CI, -5.30 to -1.92%, *P*<0.01, I^2^ = 0%) in patients with type 2 diabetes, while with no effect on TAR in patients with type 1 diabetes (**Table 2** and **Supplementary Figure S5**). Physical activity was not associated with a significant change in TBR in patients with type 1 diabetes, or type 2 diabetes (**Table 2** and **Supplementary Figure S6**).

### Meta-Regression of the Associated Factors and TIR, MAGR, TAR, and TBR

According to meta-regression analysis, patients with higher baseline BMI was associated with greater decrease in MAGE during physical activity (β=-0.392, 95% CI: -0.710, -0.074). And patients with lower baseline HbA1c levels was associated with greater increase in TBR during physical activity (β=-0.903, 95% CI: -1.550, -0.255) (**Table 3** and **Supplementary Figures S7–S10**).

**Table 3:** Meta-regression analysis for the associated factors and change in TIR, MAGE, TAR, and TBR.

Variables	Meta regression analysis	
**Time in range**	**β**	**95%CI**
Baseline HbA1c	-0.015	-0.112, 0.082
Baseline BMI	-0.006	-0.170, 0.158
Baseline age	0.005	-0.025, 0.034
Baseline male percentage	-0.009	-0.086, 0.068
Disease duration	-0.240	-0.864, 0.385
**Time above range**	**β**	**95%CI**
Baseline HbA1c	0.358	-0.066, 0.783
Baseline BMI	0.003	-0.101, 0.106
Baseline male percentage	-0.007	-0.016, 0.003
Disease duration	0.028	-0.024, 0.079
**Time below range**	**β**	**95%CI**
Baseline HbA1c	-0.903*	-1.550, -0.255
Baseline BMI	-0.015	-0.689, 0.660
Baseline age	0.040	-0.002, 0.082
Baseline male percentage	0.030	-0.011, 0.071
Disease duration	-0.124	-0.267, 0.019
**MAGE**	**β**	**95%CI**
Baseline HbA1c	-2.347	-5.482, 0.789
Baseline BMI	-0.392*	-0.710, -0.074
Baseline age	-0.058	-0.205, 0.088
Baseline male percentage	0.021	-0.017, 0.059
Disease duration	0.008	-0.325, 0.342

*p value < 0.05; HbA1c, Hemoglobin A1c; BMI, body mass index; TIR, time in range; TAR, time above range; TBR, time below range; MAGE, mean amplitude of glycemic excursion; CI, confidential interval.

## Discussion

By analyzing data from RCTs of physical activity treatment in patients with diabetes, we found that when compared with the control group, physical activity intervention was associated with significantly increased TIR and significantly decreased MAGE and TAR in patients with diabetes, but was not associated with an increase in TBR. Furthermore, patients with higher baseline BMI levels tended to have a greater decrease in MAGE during physical activities. Moreover, patients with lower baseline HbA1c levels showed a more significant increase in TBR during physical activities.

It was well known that physical activity treatment could improve glycemic control (4, 5), and our results further unveiled that physical activity might improve glycemic control by reducing the time that patients stay in hyperglycemic statues and increasing the time within the normal range. In addition to overall glycemic control, it also suggested that physical activity was associated with significantly decreased within-day glycemic variability. There were systemic reviews that also suggested that physical activity might be associated with a reduction in glycemic variability, but those reviews did not perform meta-analysis to synthesize the pooled effect of physical activity on glycemic variability and failed to conclude (42, 43). In this study, we took a step forward to quantitatively analyze the pooled effect of physical activity on glycemic variability and provided new evidence to the assumption that physical activity might reduce glycemic variability.

Concerns of hypoglycemia events have cast a shadow over the application of physical activity treatment in the clinical management of diabetes. And according to our analysis, generally speaking, physical activity would not significantly increase the time patients spent in hypoglycemia. However, we found a significant increase in TBR in patients with type 2 diabetes, and the sample size of type 1 diabetes was too small to conclude. Furthermore, patients with lower HbA1c levels tended to have more increments in TBR during physical activity. Hence, close glucose monitoring before, during, and after physical activity is crucial to preventing hypoglycemia, especially in patients with good glycemic control.

We also tried to explore which kind of patient was more suitable for physical activity treatment. Results from this study indicated that in type 2 diabetes, patients with higher baseline BMI level and short disease duration might get more reduction in glycemic variability through physical activity treatment. It might be assumed that physical activity may improve glycemic control by alleviating insulin resistance. At the early stage of type 2 diabetes, patients may have sufficient islet function, and insulin resistance tends to be the leading cause of hyperglycemia, especially in obese patients (44–46). Thus, these patients could benefit more from physical activity. However, no significant improvement in glycemic variability was found in patients with long disease duration or patients with type 1 diabetes, which might suggest that patients with poor islet function might have limited benefit in glycemic control from physical activity. Nevertheless, this hypothesis still needs further evidence to validate.

Several factors could potentially affect the effect of physical activity on glycemic variability. First of all, compensatory eating after physical activity would diminish the beneficial effect of physical activity. To evaluate the influence of compensatory eating on our results, we summarized the diet status of included studies (**Table 1**). According to the result, 11 over 13 included studies provided their participants with a standard diet during the experiment. Hence, in this study, compensatory eating after physical activity was unlikely to post a substantial influence on the beneficial effect of physical activity. Another important influencing factor was the adherence to the exercise plan. The mean adherence of patients in the included studies was summarized in **Supplementary Table S2**. Among 13 studies, 6 studies reported 99%-100% adherence of patients to the exercise plan; 1 study reported 65%-70% adherence to the exercise plan, and 6 studies did not report the adherence of patients. Thus, poor adherence of patients would probably affect the outcomes by lessening the beneficial effect of physical activity. The frequency and the intensity of physical activity would also alter the effect of physical activity on glycemic control (47). The detailed exercise plan of included studies was displayed in **Supplementary Table S2**. However, it was difficult to identify how the exercise frequency and intensity affected the effect of physical activity based on existing data. There were other characteristics, such as medication usage and comorbidities that might interfere with the glycemic control outcomes. Despite we attempted to extract and adjust these variables, they were not commonly reported in the studies. Further investigations were required to identify the influence of these variables on the effect of physical activity on glycemic variability.

Our study has its own contributions to the field. According to our knowledge, this was the first systematic review and meta-analysis to assess the relationship between physical activity and glycemic variability in patients with diabetes. Moreover, our results indicated that physical activity was associated with a significant reduction in glycemic variability, which might promote physical activity therapy in clinical practice. Several limitations should also be noted. Firstly, the sample size in type 1 diabetes was too small to draw a definite conclusion. Secondly, the definition of hypoglycemia and hyperglycemia was slightly different from trials. We summarized the definition of hypoglycemia and hyperglycemia of each trial in **Table 1**. Differences in the definition of targeted range might affect the absolute value of TIR, TAR, and TBR, which warrants caution in interpreting the results of this meta-analysis. Thirdly, the heterogeneity of studies included in the analysis of MAGE and TBR was high. We assumed two possible reasons for the heterogeneity in this study. The first one was the diversity in baseline characteristics of the included patients among trials. In subgroup analysis, the heterogeneity of MAGE decreased when stratified by baseline age and baseline BMI level. This phenomenon might partly confirm our consumption. The second one might be the variable types of physical activity and different intensity of physical activity taking in the included studies. We summarized the detailed exercise information and the compliance of participants in **Supplementary Table S2**. Differences in the type and intensity of physical activity would probably introduce heterogeneity to the analysis. Finally, most studies included in the analysis only report the glycemic data within 24 hours. Hence, it was difficult to access the between-day glycemic variability in this study. In the future, studies with a larger sample size especially for patients with type 1 diabetes, with longer intervention duration, with well designs to control the aforementioned confounding factors are required to further explore the effect of physical activity on glycemic variability.

## Conclusions

According to our study, physical activity intervention was significantly associated with decreased glycemic variability in patients with diabetes. Patients with higher BMI level and shorter disease duration might benefit more from physical activity therapy. Overall, physical activity intervention was not associated with increased time in hypoglycemia. However, hypoglycemic events related to physical activity treatment still warranted caution, especially in patients with intensive glycemic control.

## Data Availability Statement

The original contributions presented in the study are included in the article/**Supplementary Material**. Further inquiries can be directed to the corresponding authors.

## Author Contributions

XZ, XC, JC, LZhan, and LJ conceptualized this study and designed the systematic review protocol. XZ and LZhao performed the study selection and data extraction. XZ, SH, FL, and XC performed the statistical analyses. XZ, CL, and XC prepared the outlines and wrote the manuscript. All authors contributed to the article and approved the submitted version.

## Funding

This work was supported by the National Natural Science Foundation of China (No.81970698 and No.81970708), Beijing Natural Science Foundation (No.7202216), and Fundamental Research Funds for the China Institute of Sport Science (Project 15-22). The funding agencies had no roles in the study design, data collection or analysis, decision to publish, or manuscript preparation.

## Conflict of Interest

LJ has received fees for lecture presentations and for consulting from Merck, Metabasis, AstraZeneca, MSD, Novartis, Roche, Eli Lilly, SanofiAventis and Takeda.

The remaining authors declare that the research was conducted in the absence of any commercial or financial relationships that could be construed as a potential conflict of interest.

## Publisher’s Note

All claims expressed in this article are solely those of the authors and do not necessarily represent those of their affiliated organizations, or those of the publisher, the editors and the reviewers. Any product that may be evaluated in this article, or claim that may be made by its manufacturer, is not guaranteed or endorsed by the publisher.

## Abbreviations

CGM, continuous glucose monitoring; TIR, time in range; TAR, time above range; TBR, time below range; MAGE, mean amplitude of glycemic excursion; RCTs, Random controlled trials; WMDs, weighted mean differences; CI, confidence intervals; HbA1c, Hemoglobin A1c; BMI, body mass index; T1DM, type 1 diabetes; T2DM, type 2 diabetes.
