# Supplementary material for: The Effect of Physical Activity on Glycemic Variability in Patients With Diabetes: A Systematic Review and Meta-Analysis of Randomized Controlled Trials
Source: Front Endocrinol (Lausanne). 2021 Nov 17;12:767152. doi: 10.3389/fendo.2021.767152 (PMC8635769; doi:10.3389/fendo.2021.767152)
Supplement: Supplementary file 1 [file DataSheet_1.pdf]

# Supplementary Materials

**Table legends**

Table S1. Full electronic search strategy for EMBASE, MEDLINE, and Cochrane Central Register of Controlled Trials

Table S2. Description of the exercise models of included random control trials

Table S3. The risk of bias for included single-arm intervention trials

Table S4. GRADE Working Group grades of evidence for included trials

**Figure legends**

Figure S1. Funnel plot of RCTs included in this meta-analysis

Figure S2. Beggs test of RCTs included in this meta-analysis

Figure S3. Comparison of TIR change between physical activity treatment group and control group stratified by disease type

Figure S4. Comparison of MAGE change between physical activity treatment group and control group stratified by disease type

Figure. S5 Comparison of TAR change between physical activity treatment group and control group stratified by disease type

Figure. S6 Comparison of TBR change between physical activity treatment group and control group stratified by disease type

Figure. S7 Associated factors with the change of TIR by meta-regression analysis. Associated factors: Baseline HbA1c (A), Baseline BMI (B), Baseline age (C), Baseline male percentage (D), and Disease duration (E)

Figure. S8 Associated factors with the change of MAGE by meta-regression analysis. Associated factors: Baseline HbA1c (A), Baseline BMI (B), Baseline age (C), Baseline male percentage (D), and Disease duration (E)

Figure. S9 Associated factors with the change of TAR by meta-regression analysis. Associated factors: Baseline HbA1c (A), Baseline BMI (B), Baseline age (C), Baseline male percentage (D), and Disease duration (E)

Figure. S10 Associated factors with the change of TBR by meta-regression analysis. Associated factors: Baseline HbA1c (A), Baseline BMI (B), Baseline age (C), Baseline male percentage (D), and Disease duration (E)

Table S1. Full electronic search strategy for EMBASE, MEDLINE, and Cochrane Central Register of Controlled Trials

| **Data Base** | **Full electronic search strategy** |
|---|---|
| EMBASE | 'muscle strength'/exp OR 'weight lifting'/exp OR 'circuit training'/exp OR 'resistance training'/exp OR 'aerobic training'/exp OR 'kinesiotherapy'/exp OR 'exercise'/exp AND 'diabetes mellitus'/exp AND 'continuous glucose monitoring'/exp |
| MEDLINE | ((("Exercise"[Mesh] OR "Circuit-Based Exercise"[Mesh] OR "Cool-Down Exercise"[Mesh] OR "Warm-Up Exercise"[Mesh] OR "Exercise Movement Techniques"[Mesh] OR "Exercise Therapy"[Mesh] OR "Exercise Test"[Mesh] OR "Resistance Training"[Mesh] OR "Muscle Stretching Exercises"[Mesh] OR "High-Intensity Interval Training"[Mesh]) AND ( "Diabetes Mellitus"[Mesh] OR "Diabetes, Gestational"[Mesh] OR "Diabetes Mellitus, Type 2"[Mesh] )) ) AND (continuous glucose monitoring) |
| Cochrane Central Register of Controlled Trials | "Diabetes Mellitus" or "Diabetes Insipidus" and "muscle strength" or "muscle strengthening" or "muscle-strengthening" or "weight lifting" or weightlifting or "weight bearing" or weight-bearing or "weight training" or "circuit training" or "strength exercise" or "strengthening exercise" or "strength training" or "resistance exercise" or "resistance training" or "progressive resistance" or "Physical Exercise" or "Isometric Exercise" or "aerobic exercise" or "aerobic training" or "exercise therapy" and "continuous glucose monitoring" |

Table S2. Description of the exercise models of included random control trials

| **Author, year** | **Exercise duration** | **Mean adherence (%)** | **Treatment group** | **Specific exercise type** | **Relative intensity values** | **Exercise time/ total sets per sessions** | **Frequency** |
|---|---|---|---|---|---|---|---|
| Paddy 2017(1) | 24 hours | 100 | Light-intensity walking | Walking | NA | Sitting plus 3 min bouts of light-intensity walking at 3.2 km/h every 30 min; | NA |
| | | | Simple resistance activities | Alternating half-squats, calf raises, brief gluteal contractions and knee raises | NA | Sitting plus 3 min bouts of simple resistance activities every 30 min | NA |
| | | | Control | prolonged sitting | NA | NA | NA |
| Jonida 2016(2) | 24hours | NA | Continous walking | Walking on the treadmill | 50 % | Continous walking 40min | 1 session per 24 hours |
| | | | Split walking | Walking on the treadmill | 50 % | Split walking 20min+20min | 1 session per 24 hours |
| | | | Control | NA | NA | NA | NA |
| Jordan 2019(3) | 24hours | 100 | Exercise | Walking on the treadmill | NA | Walking at 5.0 km/hour and 0.5% | 1 session per 24 |

| | | | | | | | |
|---|---|---|---|---|---|---|---|
| | | | | | | incline; A 5-min warm up and cooldown at a pace of 3.5 km/hour and 0.0% grade were included | hours |
| | | | Control | 50 min of sitting | NA | NA | 1 session per 24 hours |
| Zheng Li 2018(4) | 24hours | 100 | Exercise | walking on a treadmill after dinner | 40% | 20min | 1 session per 24 hour |
| | | | control | NA | NA | NA | NA |
| Richard 2018(5) | 24hours | 100 | Reduced-exertion high-intensity interval training (REHIT) | performed on mechanically braked cycle ergometer | Exercise at a resistance equivalent to 5% of body mass | 10 × 60 s cycling efforts interspersed with 60 s of low-intensity recovery | 1 session per 24 hours |
| | | | Moderate–vigorous-intensity continuous exercise 30 min (MICT) | performed on an electronically braked cycle ergometer | 50% | 30 min of continuous cycling a | 1 session per 24 hours |
| | | | High-intensity | performed on an | 85% | 10 × 60 s cycling | 1 session |

| | | | | | | | |
|---|---|---|---|---|---|---|---|
| | | | interval training (HIIT) | electronically braked cycle ergometer | | efforts interspersed with 60 s of low-intensity recovery | per 24 hours |
| | | | Control | NA | NA | NA | NA |
| Kristian 2017(6) | 2weeks | 99% | Continuous walking | Walking on a treadmill | 73% | 60min | 5 days per week |
| | | | interval walking training | Walking on a treadmill | 54% and 89% | 60min, alternating cycles of 3 min slow walking (54% of $\dot{V}O_2$ peak) and 3 min fast walking (89% of VO 2peak ). | 5 days per week |
| | | | control | NA | NA | NA | NA |
| Myette-Côté 2016(7) | 24 hours | NA | Exercise | Walking | NA | 50 minutes | 1 session per 24 hours |
| | | | control | NA | NA | NA | NA |
| Tasuku 2016(8) | 24hours | NA | Moderate-intensity continuous exercise | Exercise on a treadmill | 55% | 60min | 1 session per 24 hours |
| | | | High-intensity interval | Exercise on a treadmill | 40%-100% | 60min (3 minutes at | 1 session per 24 |

| | | | | | | | |
|---|---|---|---|---|---|---|---|
| | | | exercise | | | workload corresponding to 40% 1minute at workload corresponding to 100% $VO_2$ peak) | hours |
| | | | Control | NA | NA | NA | NA |
| Jennifer 2019(9) | 24h | 100% | morning walk after breakfast | continuous brisk walk | NA | 20min, 40min, and 60min | 1 session per 24 hours |
| | | | post-meal breaks from sitting | short physical activity | NA | short physical activity | NA |
| | | | control | NA | NA | NA | NA |
| Matthew 2020(10) | 12 days | NA | Morning exercise | Walking on a treadmill at 5.0 km/h with 0.5% grade. | NA | 40 minutes at 5.0 km/h with 0.5% grade. | 1session per24hours |
| | | | Afternoon exercise | Walking on a treadmill at 5.0 km/h with 0.5% grade. | NA | 40 minutes at 5.0 km/h with 0.5% grade. | 1session per24hours |
| | | | Evening exercise | Walking on a treadmill at 5.0 km/h with 0.5% | NA | 40 minutes at 5.0 km/h with 0.5% grade. | 1session per24hours |

| | | | | | | | |
|---|---|---|---|---|---|---|---|
| | | | | grade. | | | |
| | | | control | NA | NA | NA | NA |
| Ravi 2019(11) | 3weeks | NA | Aerobic exercise | Treadmill exercise | 60% | 45min | 2sessions per week |
| | | | resistance exercise | five different exercises (leg press, bench press, leg extension, leg flexion and seated row) | 60-80% | three sets of 8–12 repetitions | 2sessions per week |
| | | | control | NA | NA | NA | NA |
| Kamilla 2018(12) | 11weeks | NA | Endurance training | Cycling | 50% | 40 min | 3 days per week |
| | | | High-intensity interval training | Cycling | 95% | 1 min intervals at 95% of peak workload interspersed by 1 min active recovery for 20 min | 3 days per week |
| | | | Control | Daily activities | NA | NA | NA |
| Angela 2020(13) | 12weeks | 67% | high-intensity interval training | cycle ergometer or treadmill | 60–95%peakheartrate | 5 min of warm-up at60%peak heartrate<br>4 bouts of 4-min high-intensity intervals at | 3 days per week |

| | | | | | | |
|---|---|---|---|---|---|---|
| | | | | | 85–95% HR peak Three bouts of 3-min recovery intervals at 50–70%HR peak | |
| | 65% | control | Daily activities | NA | NA | NA |

*HR: heart rate; NA: not available;*

Table S3. The risk of bias for included random control trials

| Author, year | Adequate randomization sequence generation | Adequate allocation concealment | Blinding of participants and caregivers | Binding of outcome assessors and adjudicators | Free of infrequent missing outcome data | Free of selective outcome reporting | Free of other bias |
|---|---|---|---|---|---|---|---|
| Paddy 2017(1) | **Probably yes** Randomized, Open label | **Probably no** Randomized, Open label | **Definitely no** Open label | **Definitely yes** | **Definitely yes** There were 0% missing outcome data; | **Definitely yes** | **Probably yes** Generally balanced baseline characteristics across groups |
| Jonida Haxhi 2016(2) | **Probably yes** Randomized, Open label | **Probably no** Randomized, Open label | **Definitely no** Open label | **Definitely yes** | **Definitely yes** There were 0% missing outcome data; | **Definitely yes** | **Probably yes** Generally balanced baseline characteristics across groups |
| Jordan L.Rees 2019(3) | **Probably yes** Randomize | **Probably no** Randomized, Open label | **Definitely no** Open | **Definitely yes** | **Probably yes** There | **Definitely yes** | **Probably yes** Generally |

| | | | | | | | |
|---|---|---|---|---|---|---|---|
| | d, Open label | | label | | were 21.1% patients with missing outcome data; The ing outcome data were generally balanced across treatment groups, with similar reasons for missing data | | balanced baseline characteristics across groups |
| Zheng Li 2018(4) | **Probably yes** Randomized, Open label | **Probably no** Randomized, Open label | **Definitely no** Open label | **Definitely yes** | **Definitely yes** There were 0% missing outcome data; | **Definitely yes** | **Probably yes** Generally balanced baseline characteristics across groups |
| Richard S.Metcalfe 2018(5) | **Probably yes** Randomized, Open label | **Probably no** Randomized, Open label | **Definitely no** Open label | **Definitely yes** | **Definitely yes** There were 0% missing outcome data; | **Definitely yes** | **Probably yes** Generally balanced baseline characteris |

| | | | | | | | |
|---|---|---|---|---|---|---|---|
| | | | | | | | tics across groups |
| Kristian Karstoft 2017(6) | **Definitely yes** Randomized, Open label Computer generated randomization | **Probably no** Randomized, Open label | **Definitely no** Open label | **Definitely yes** | **Probably yes** There were 9.1% (1/11) patients with missing outcome data; The missing outcome data were generally balanced across treatment groups, with similar reasons for missing data | **Definitely yes** | **Probably yes** Generally balanced baseline characteristics across groups |
| Myette-Côté 2016(7) | **Probably yes** Randomized, Open label | **Probably no** Randomized, Open label | **Definitely no** Open label | **Definitely yes** | **Definitely yes** There were 0% missing outcome data; | **Definitely yes** | **Probably yes** Generally balanced baseline characteristics across groups |
| Tasuku | **Probably** | **Probably no** | **Definitely** | **Definitely yes** | **Definitely** | **Definitely** | **Probably** |

| | | | | | | | |
|---|---|---|---|---|---|---|---|
| 2016(8) | **yes** Randomized, Open label | Randomized, Open label | **no** Open label | | **yes** There were 0% missing outcome data; | **yes** | **yes** Generally balanced baseline characteristics across groups |
| Jennifer 2019(9) | **Probably yes** Randomized, Open label | **Probably no** Randomized, Open label | **Definitely no** Open label | **Definitely yes** | **Definitely yes** There were 0% missing outcome data; | **Definitely yes** | **Probably yes** Generally balanced baseline characteristics across groups |
| Matthew 2020(10) | **Probably yes** Randomized, Open label a Latin square | **Probably no** Randomized, Open label | **Definitely no** Open label | **Definitely yes** | **Probably yes** There were 21.4% (3/14) patients with missing outcome data; The missing outcome data were generally balanced across treatment | **Definitely yes** | **Probably yes** Generally balanced baseline characteristics across groups |

| | | | | | | | |
|---|---|---|---|---|---|---|---|
| | | | | | groups, with similar reasons for missing data | | |
| Ravi Reddy 2019(11) | **Probably yes** Randomized, Open label | **Probably no** Randomized, Open label | **Definitely no** Open label | **Definitely yes** | **Definitely yes** There were 0% missing outcome data; | **Definitely yes** | **Probably yes** Generally balanced baseline characteristics across groups |
| Kamilla 2018(12) | **Definitely yes** Randomized, Open label sealed envelopes | **Probably no** Randomized, Open label | **Definitely no** Open label | **Definitely yes** | **Definitely yes** There were 0% missing outcome data; | **Definitely yes** | **Probably yes** Generally balanced baseline characteristics across groups |
| Angela 2020(13) | **Probably yes** Randomized, Open label | **Probably no** Randomized, Open label | **Definitely no** Open label | **Definitely yes** | **Probably yes** There were 16.7% (2/12) and 20% (3/15) patients in exercise and | **Definitely yes** | **Probably yes** Generally balanced baseline characteristics across |

| | | |
|---|---|---|
| | control groups with missing outcome data, respectively; missing outcome data were generally balanced across treatment groups, with similar reasons for missing data | groups |

Table S4. GRADE Working Group grades of evidence for included trials

| Quality assessment | | | | | | | No of patients | | Effect | | Quality | Importance |
|---|---|---|---|---|---|---|---|---|---|---|---|---|
| No of studies | Design | Risk of bias | Inconsistency | Indirectness | Imprecision | Other considerations | Physical activity | Control | Relative (95% CI) | Absolute | | |
| **MAGE (Better indicated by lower values)** | | | | | | | | | | | | |
| 19 | randomised trials | serious[1] | serious[2] | no serious indirectness | no serious imprecision | none | 303 | 311 | - | MD 0.68 lower (1.01 to 0.36 lower) | ⊕⊕OO LOW | not available |
| **TIR (Better indicated by lower values)** | | | | | | | | | | | | |
| 6 | randomised trials | serious[3] | no serious inconsistency | no serious indirectness | no serious imprecision | none | 120 | 112 | - | MD 4.17 higher (1.11 to 7.23 higher) | ⊕⊕⊕O MODERATE | not available |
| **TAR (Better indicated by lower values)** | | | | | | | | | | | | |
| 19 | randomised trials | serious[3] | no serious inconsistency | no serious indirectness | no serious imprecision | none | 265 | 265 | - | MD 3.54 lower (5.21 to 1.88 lower) | ⊕⊕⊕O MODERATE | not available |
| **TBR (Better indicated by lower values)** | | | | | | | | | | | | |
| 8 | randomised trials | serious[3] | serious | no serious indirectness | no serious imprecision | none | 95 | 87 | - | MD 1.54 higher (0.24 lower to 3.32 higher) | ⊕⊕OO LOW | not available |

[1] Lack of blinding

[2] Unexplained high heterogeneity of results

[3] Unexplained heterogeneity of results

**High quality:** Further research is very unlikely to change our confidence in the estimate of effect.

**Moderate quality:** Further research is likely to have an important impact on our confidence in the estimate of effect and may change the estimate.

**Low quality:** Further research is very likely to have an important impact on our confidence in the estimate of effect and is likely to change the estimate.

**Very low quality:** We are very uncertain about the estimate.

Figure S1. Funnel plot of RCTs included in this meta-analysis

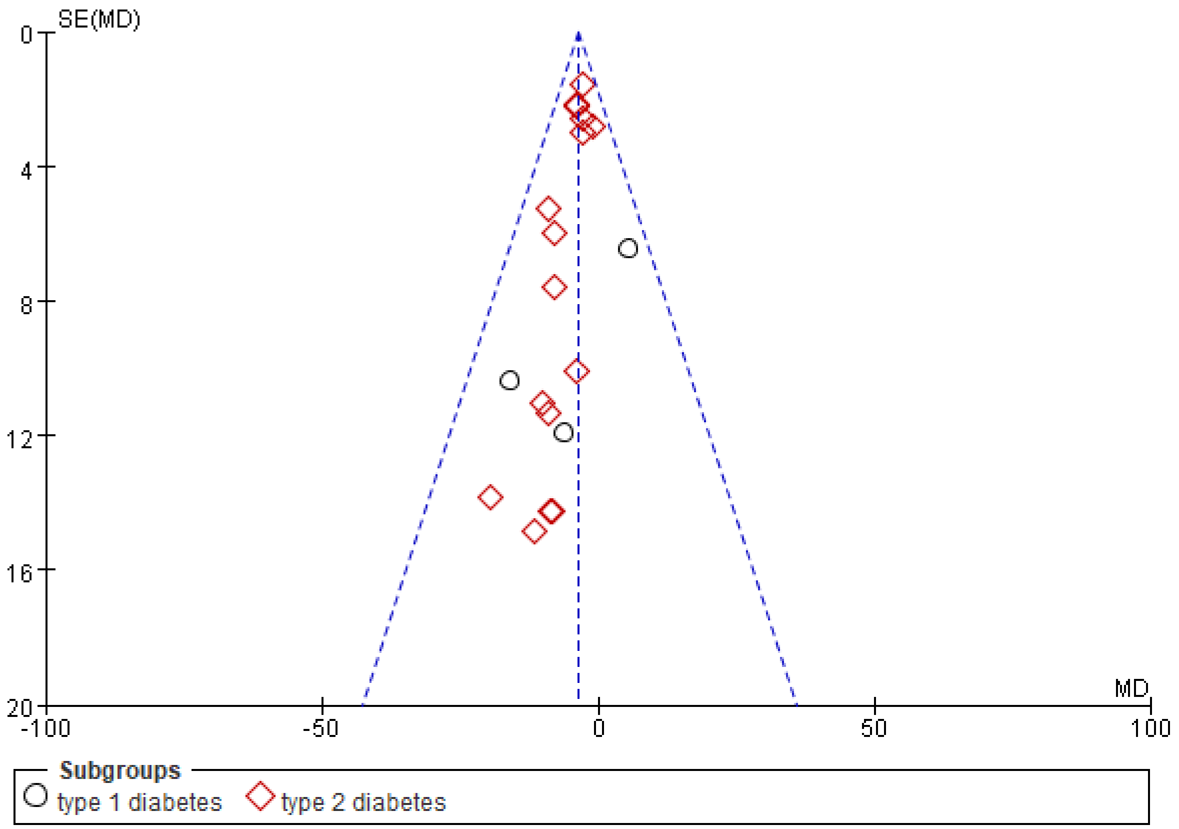
SE(MD)
0
4
8
12
16
20
MD
-100
-50
0
50
100
Subgroups
type 1 diabetes
type 2 diabetes

Figure S2. Beggs test of RCTs included in this meta-analysis

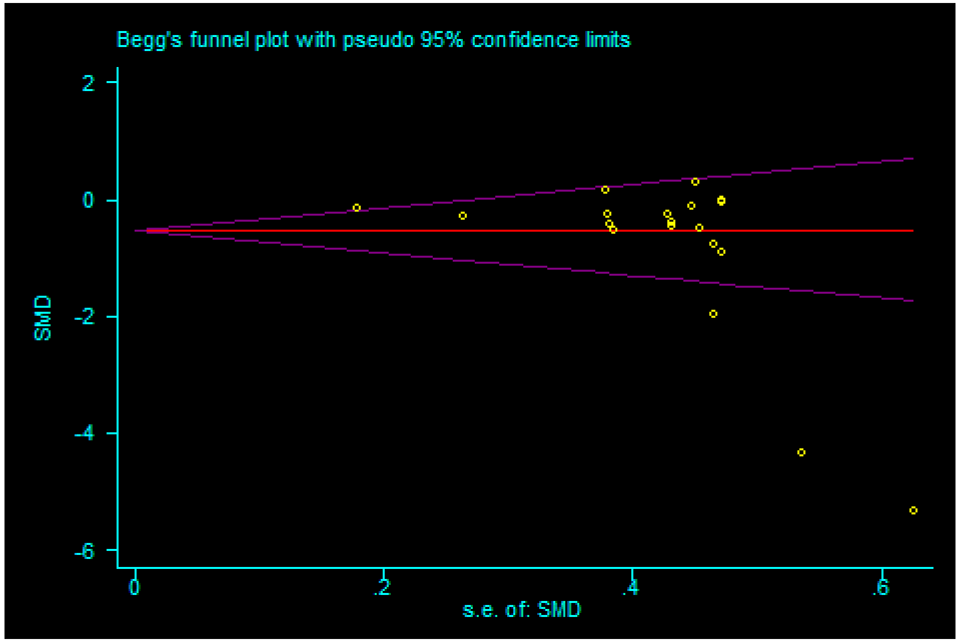

Figure S3. Comparison of TIR change between physical activity treatment group and control group stratified by disease type

| Study or Subgroup | Exercise Mean [%] | Exercise SD [%] | Exercise Total | Control Mean [%] | Control SD [%] | Control Total | Weight | Mean Difference IV, Fixed, 95% CI [%] |
|---|---|---|---|---|---|---|---|---|
| **3.4.1 type 1 diabetes** | | | | | | | | |
| Angela 2020 | 45.5 | 17.6 | 12 | 46.5 | 12.5 | 15 | 6.7% | -1.00 [-12.80, 10.80] |
| Ravi 2018(Aerobic exercise) | 60.5 | 22 | 10 | 55.7 | 25 | 10 | 2.2% | 4.80 [-15.84, 25.44] |
| Ravi 2018(Resistance exercise) | 70.3 | 15 | 10 | 55.7 | 25 | 10 | 2.9% | 14.60 [-3.47, 32.67] |
| **Subtotal (95% CI)** | | | **32** | | | **35** | **11.8%** | **3.87 [-5.04, 12.78]** |
| Heterogeneity: Chi² = 2.02, df = 2 (P = 0.36); I² = 1% | | | | | | | | |
| Test for overall effect: Z = 0.85 (P = 0.39) | | | | | | | | |
| **3.4.2 type 2 diabetes** | | | | | | | | |
| Jordan 2019 | 95 | 8.5 | 63 | 91 | 10.5 | 63 | 84.2% | 4.00 [0.66, 7.34] |
| Kamilla 2018 (Endurance training) | 86 | 17 | 12 | 77 | 26 | 7 | 2.0% | 9.00 [-12.53, 30.53] |
| Kamilla 2018 (High-intensity interval training) | 85 | 18 | 13 | 77 | 26 | 7 | 2.0% | 8.00 [-13.60, 29.60] |
| **Subtotal (95% CI)** | | | **88** | | | **77** | **88.2%** | **4.21 [0.95, 7.46]** |
| Heterogeneity: Chi² = 0.32, df = 2 (P = 0.85); I² = 0% | | | | | | | | |
| Test for overall effect: Z = 2.53 (P = 0.01) | | | | | | | | |
| **Total (95% CI)** | | | **120** | | | **112** | **100.0%** | **4.17 [1.11, 7.23]** |
| Heterogeneity: Chi² = 2.35, df = 5 (P = 0.80); I² = 0% | | | | | | | | |
| Test for overall effect: Z = 2.67 (P = 0.008) | | | | | | | | |
| Test for subgroup differences: Chi² = 0.00, df = 1 (P = 0.95), I² = 0% | | | | | | | | |

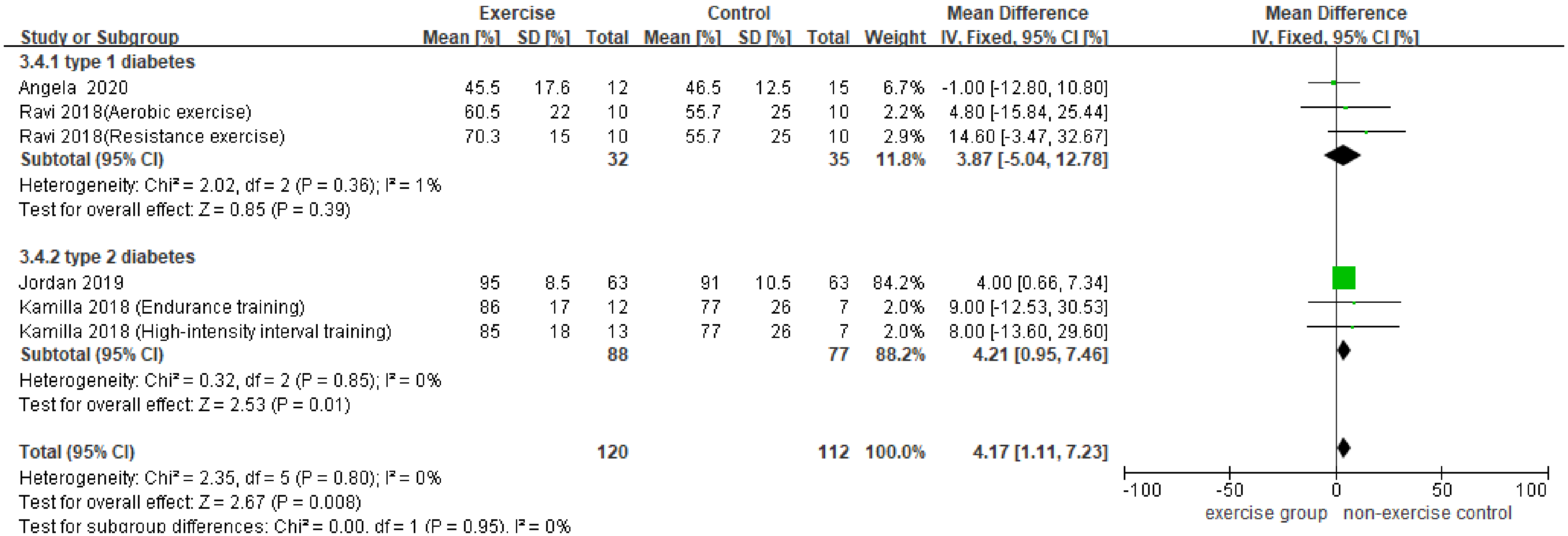


*CI: confidential interval;*

Figure S4. Comparison of MAGE change between physical activity treatment group and control group stratified by disease type

| Study or Subgroup | Exercise Mean [mmol/L] | Exercise SD [mmol/L] | Exercise Total | Control Mean [mmol/L] | Control SD [mmol/L] | Control Total | Weight | Mean Difference IV, Random, 95% CI [mmol/L] |
|---|---|---|---|---|---|---|---|---|
| **3.1.2 type 2 diabetes** | | | | | | | | |
| Jonida 2015(Continuous walking) | 3.27 | 2.37 | 9 | 3.39 | 1.36 | 9 | 2.5% | -0.12 [-1.91, 1.67] |
| Jonida Haxhi 2015(Split walking) | 3.39 | 1.14 | 9 | 3.39 | 1.36 | 9 | 4.6% | 0.00 [-1.16, 1.16] |
| Jordan 2019 | 4.2 | 1.8 | 63 | 4.5 | 2.2 | 63 | 7.2% | -0.30 [-1.00, 0.40] |
| Kristian 2016 (Continuous walking training) | 6.5 | 0.7 | 14 | 6.4 | 0.6 | 14 | 8.7% | 0.10 [-0.38, 0.58] |
| Kristian 2016 (Interval walking training) | 5.4 | 0.4 | 14 | 6.4 | 0.6 | 14 | 9.4% | -1.00 [-1.38, -0.62] |
| Matthew 2020 (Afternoon Exercise) | 3.7 | 1.1 | 14 | 4.3 | 1.2 | 14 | 6.2% | -0.60 [-1.45, 0.25] |
| Matthew 2020 (Evening Exercise) | 3.8 | 1.2 | 14 | 4.3 | 1.2 | 14 | 6.0% | -0.50 [-1.39, 0.39] |
| Matthew 2020 (Morning Exercise) | 4 | 1.3 | 14 | 4.3 | 1.2 | 14 | 5.8% | -0.30 [-1.23, 0.63] |
| Myette-Côté 2015 | 3.9 | 1.7 | 10 | 3.4 | 1.5 | 10 | 3.6% | 0.50 [-0.91, 1.91] |
| Paddy 2017(Light-intensity walking) | 4.6 | 0.3 | 24 | 5.9 | 0.3 | 24 | 10.4% | -1.30 [-1.47, -1.13] |
| Paddy 2017(Simple resistance activities) | 4.3 | 0.3 | 24 | 5.9 | 0.3 | 24 | 10.4% | -1.60 [-1.77, -1.43] |
| Richard 2018 (HIIT) | 3.42 | 1.5 | 11 | 4.21 | 2.04 | 11 | 3.3% | -0.79 [-2.29, 0.71] |
| Richard 2018 (MICT) | 3.47 | 1.59 | 11 | 4.21 | 2.04 | 11 | 3.2% | -0.74 [-2.27, 0.79] |
| Richard 2018(REHIT) | 3.76 | 1.35 | 11 | 4.21 | 2.04 | 11 | 3.5% | -0.45 [-1.90, 1.00] |
| Tasuku 2016(Fasting HIIT) | 3.24 | 2.01 | 9 | 5.03 | 2.01 | 10 | 2.5% | -1.79 [-3.60, 0.02] |
| Tasuku 2016(Fasting MICT) | 3.49 | 2.01 | 8 | 5.03 | 2.01 | 10 | 2.4% | -1.54 [-3.41, 0.33] |
| Tasuku 2016(Feeding HIIT) | 4.77 | 2.01 | 7 | 5.03 | 2.01 | 10 | 2.2% | -0.26 [-2.20, 1.68] |
| Tasuku 2016(Feeding MICT) | 4.05 | 2.01 | 8 | 5.03 | 2.01 | 10 | 2.4% | -0.98 [-2.85, 0.89] |
| Zheng Li 2018 | 2.6 | 1.9 | 29 | 3.1 | 1.6 | 29 | 5.9% | -0.50 [-1.40, 0.40] |
| **Subtotal (95% CI)** | | | **303** | | | **311** | **100.0%** | **-0.68 [-1.01, -0.36]** |
| Heterogeneity: Tau² = 0.26; Chi² = 82.23, df = 18 (P < 0.00001); I² = 78% | | | | | | | | |
| Test for overall effect: Z = 4.10 (P < 0.0001) | | | | | | | | |
| **Total (95% CI)** | | | **303** | | | **311** | **100.0%** | **-0.68 [-1.01, -0.36]** |
| Heterogeneity: Tau² = 0.26; Chi² = 82.23, df = 18 (P < 0.00001); I² = 78% | | | | | | | | |
| Test for overall effect: Z = 4.10 (P < 0.0001) | | | | | | | | |
| Test for subgroup differences: Not applicable | | | | | | | | |

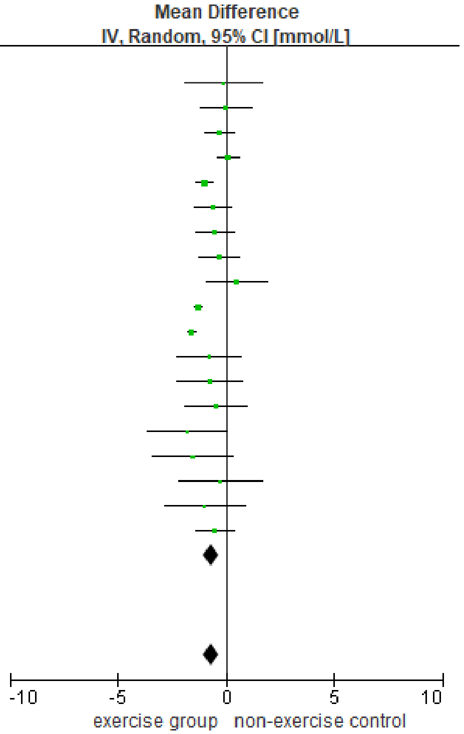


*CI: confidential interval;*

Figure. S5 Comparison of TAR change between physical activity treatment group and control group stratified by disease type

| | Exercise | | | Control | | | | Mean Difference |
|---|---|---|---|---|---|---|---|---|
| Study or Subgroup | Mean [%] | SD [%] | Total | Mean [%] | SD [%] | Total | Weight | IV, Fixed, 95% CI [%] |
| **3.5.1 type 1 diabetes** | | | | | | | | |
| Angela 2020 | 48.6 | 17.4 | 12 | 43 | 15.6 | 15 | 1.7% | 5.60 [-7.02, 18.22] |
| Ravi 2018(Aerobic exercise) | 32.9 | 25 | 10 | 39.1 | 28 | 10 | 0.5% | -6.20 [-29.47, 17.07] |
| Ravi 2018(Resistance exercise) | 23.1 | 17 | 10 | 39.1 | 28 | 10 | 0.7% | -16.00 [-36.30, 4.30] |
| **Subtotal (95% CI)** | | | **32** | | | **35** | **2.9%** | **-1.43 [-11.17, 8.30]** |
| Heterogeneity: Chi² = 3.33, df = 2 (P = 0.19); I² = 40% | | | | | | | | |
| Test for overall effect: Z = 0.29 (P = 0.77) | | | | | | | | |
| **3.5.2 type 2 diabetes** | | | | | | | | |
| Jordan 2019 | 4 | 8.5 | 63 | 7 | 9 | 63 | 29.6% | -3.00 [-6.06, 0.06] |
| Kamilla 2018 (Endurance training) | 13 | 17 | 12 | 23 | 26 | 7 | 0.6% | -10.00 [-31.53, 11.53] |
| Kamilla 2018 (High-intensity interval training) | 14 | 20 | 13 | 23 | 26 | 7 | 0.6% | -9.00 [-31.12, 13.12] |
| Kristian 2016 (Continuous walking training) | 18.6 | 4.9 | 14 | 22.4 | 6.5 | 14 | 15.2% | -3.80 [-8.06, 0.46] |
| Kristian 2016 (Interval walking training) | 18.6 | 5.1 | 14 | 22.4 | 6.5 | 14 | 14.8% | -3.80 [-8.13, 0.53] |
| Matthew 2020 (Afternoon Exercise) | 6 | 8 | 14 | 9 | 7.8 | 14 | 8.1% | -3.00 [-8.85, 2.85] |
| Matthew 2020 (Evening Exercise) | 8 | 7 | 14 | 9 | 7.8 | 14 | 9.2% | -1.00 [-6.49, 4.49] |
| Matthew 2020 (Morning Exercise) | 6 | 5.6 | 14 | 9 | 7.8 | 14 | 11.0% | -3.00 [-8.03, 2.03] |
| Myette-Côté 2015 | 18 | 21 | 10 | 22 | 24 | 10 | 0.7% | -4.00 [-23.77, 15.77] |
| Richard 2018 (HIIT) | 16 | 9 | 11 | 25 | 15 | 11 | 2.6% | -9.00 [-19.34, 1.34] |
| Richard 2018 (MICT) | 17 | 20 | 11 | 25 | 15 | 11 | 1.3% | -8.00 [-22.77, 6.77] |
| Richard 2018(REHIT) | 17 | 13 | 11 | 25 | 15 | 11 | 2.0% | -8.00 [-19.73, 3.73] |
| Tasuku 2016(Fasting HIIT) | 14.1 | 30 | 9 | 33.75 | 30 | 10 | 0.4% | -19.65 [-46.67, 7.37] |
| Tasuku 2016(Fasting MICT) | 25.42 | 30 | 8 | 33.75 | 30 | 10 | 0.4% | -8.33 [-36.22, 19.56] |
| Tasuku 2016(Feeding HIIT) | 22.15 | 30 | 7 | 33.75 | 30 | 10 | 0.3% | -11.60 [-40.58, 17.38] |
| Tasuku 2016(Feeding MICT) | 24.93 | 30 | 8 | 33.75 | 30 | 10 | 0.4% | -8.82 [-36.71, 19.07] |
| **Subtotal (95% CI)** | | | **233** | | | **230** | **97.1%** | **-3.61 [-5.30, -1.92]** |
| Heterogeneity: Chi² = 5.51, df = 15 (P = 0.99); I² = 0% | | | | | | | | |
| Test for overall effect: Z = 4.19 (P < 0.0001) | | | | | | | | |
| **Total (95% CI)** | | | **265** | | | **265** | **100.0%** | **-3.54 [-5.21, -1.88]** |
| Heterogeneity: Chi² = 9.03, df = 18 (P = 0.96); I² = 0% | | | | | | | | |
| Test for overall effect: Z = 4.17 (P < 0.0001) | | | | | | | | |
| Test for subgroup differences: Chi² = 0.19, df = 1 (P = 0.67), I² = 0% | | | | | | | | |

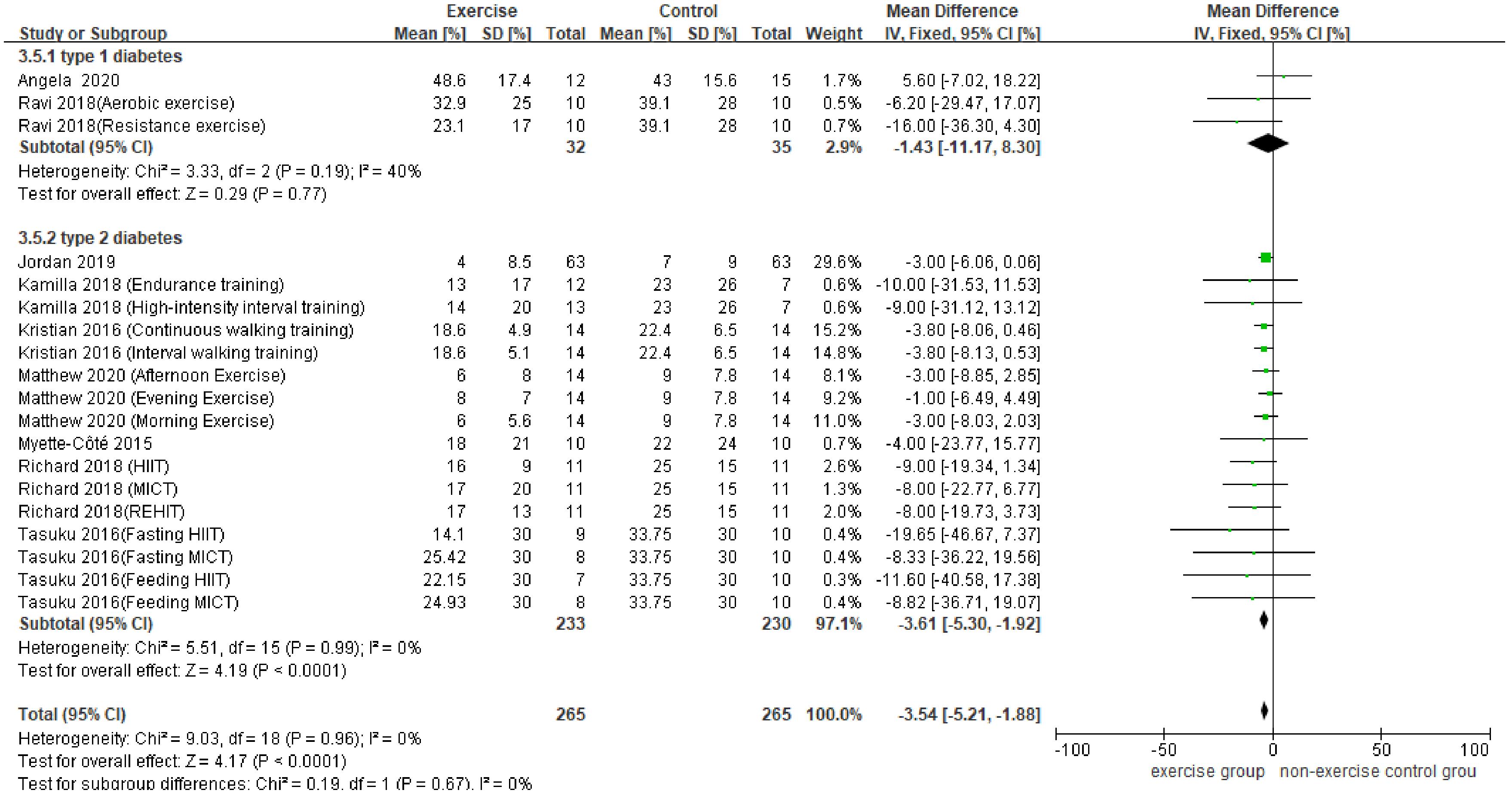


*TAR: time above range; CI: confidential interval;*

Figure. S6 Comparison of TBR change between physical activity treatment group and control group stratified by disease type

| Study or Subgroup | Exercise Mean [%] | Exercise SD [%] | Exercise Total | Control Mean [%] | Control SD [%] | Control Total | Weight | Mean Difference IV, Random, 95% CI [%] |
|---|---|---|---|---|---|---|---|---|
| **3.6.1 type 1 diabetes** | | | | | | | | |
| Angela 2020 | 5.9 | 4 | 12 | 10.5 | 9.4 | 15 | 8.4% | -4.60 [-9.87, 0.67] |
| Ravi 2018(Aerobic exercise) | 3.17 | 9.83 | 10 | 1.86 | 7.15 | 10 | 5.5% | 1.31 [-6.22, 8.84] |
| Ravi 2018(Resistance exercise) | 3.63 | 6.07 | 10 | 1.86 | 7.15 | 10 | 7.5% | 1.77 [-4.04, 7.58] |
| **Subtotal (95% CI)** | | | **32** | | | **35** | **21.4%** | **-0.89 [-5.22, 3.44]** |
| Heterogeneity: Tau² = 5.00; Chi² = 3.03, df = 2 (P = 0.22); I² = 34% | | | | | | | | |
| Test for overall effect: Z = 0.40 (P = 0.69) | | | | | | | | |
| **3.6.2 type 2 diabetes** | | | | | | | | |
| Kamilla 2018 (Endurance training) | 1 | 2 | 12 | 0 | 0.001 | 7 | 16.1% | 1.00 [-0.13, 2.13] |
| Kamilla 2018 (High-intensity interval training) | 1 | 3 | 13 | 0 | 0.001 | 7 | 15.4% | 1.00 [-0.63, 2.63] |
| Kristian 2016 (Continuous walking training) | 5.3 | 2.2 | 14 | 2.8 | 1.2 | 14 | 15.8% | 2.50 [1.19, 3.81] |
| Kristian 2016 (Interval walking training) | 0 | 0.001 | 14 | 2.8 | 1.2 | 14 | 16.6% | -2.80 [-3.43, -2.17] |
| Myette-Côté 2015 | 2.1 | 3.2 | 10 | 0.1 | 0.3 | 10 | 14.7% | 2.00 [0.01, 3.99] |
| **Subtotal (95% CI)** | | | **63** | | | **52** | **78.6%** | **0.69 [-1.78, 3.15]** |
| Heterogeneity: Tau² = 7.41; Chi² = 85.05, df = 4 (P < 0.00001); I² = 95% | | | | | | | | |
| Test for overall effect: Z = 0.55 (P = 0.58) | | | | | | | | |
| **Total (95% CI)** | | | **95** | | | **87** | **100.0%** | **0.36 [-1.79, 2.51]** |
| Heterogeneity: Tau² = 7.13; Chi² = 88.08, df = 7 (P < 0.00001); I² = 92% | | | | | | | | |
| Test for overall effect: Z = 0.33 (P = 0.74) | | | | | | | | |
| Test for subgroup differences: Chi² = 0.38, df = 1 (P = 0.54), I² = 0% | | | | | | | | |

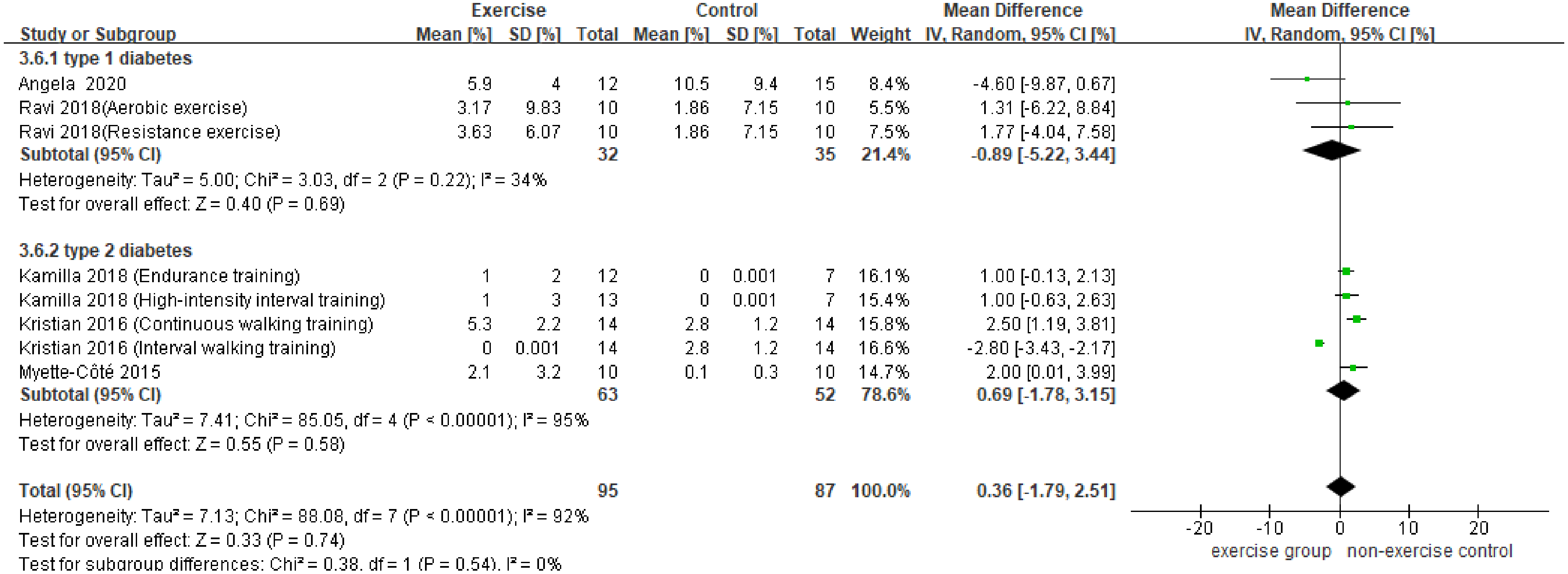


*TBR: time below range; CI: confidential interval;*

Figure. S7 Associated factors with the change of TIR by meta-regression analysis. Associated factors: Baseline HbA1c (A), Baseline BMI (B), Baseline age (C), Baseline male percentage (D), and Disease duration (E)

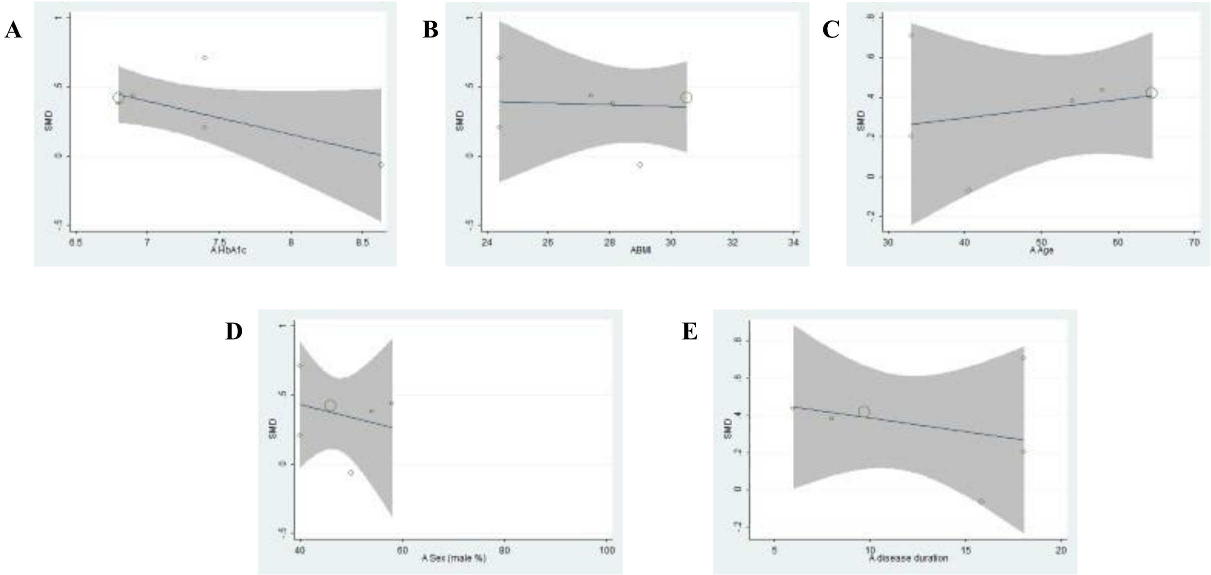


*HbA1c: Hemoglobin A1c; BMI: body mass index; TIR: time in range;*

Figure. S8 Associated factors with the change of MAGE by meta-regression analysis. Associated factors: Baseline HbA1c (A), Baseline BMI (B), Baseline age (C), Baseline male percentage (D), and Disease duration (E)

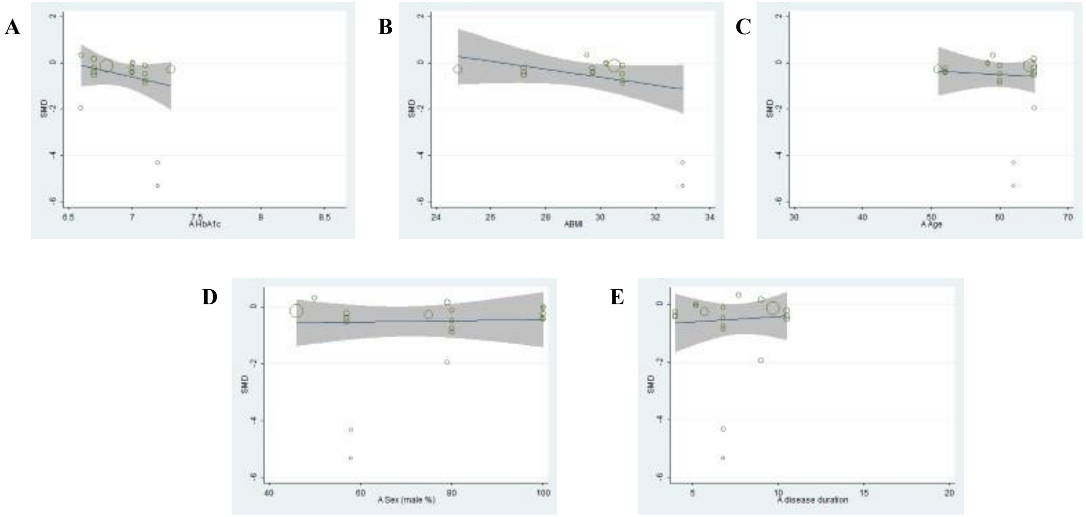

*HbA1c: Hemoglobin A1c; BMI: body mass index; MAGE: mean amplitude of glycemic excursion;*

Figure. S9 Associated factors with the change of TAR by meta-regression analysis. Associated factors: Baseline HbA1c (A), Baseline BMI (B), Baseline age (C), Baseline male percentage (D), and Disease duration (E)

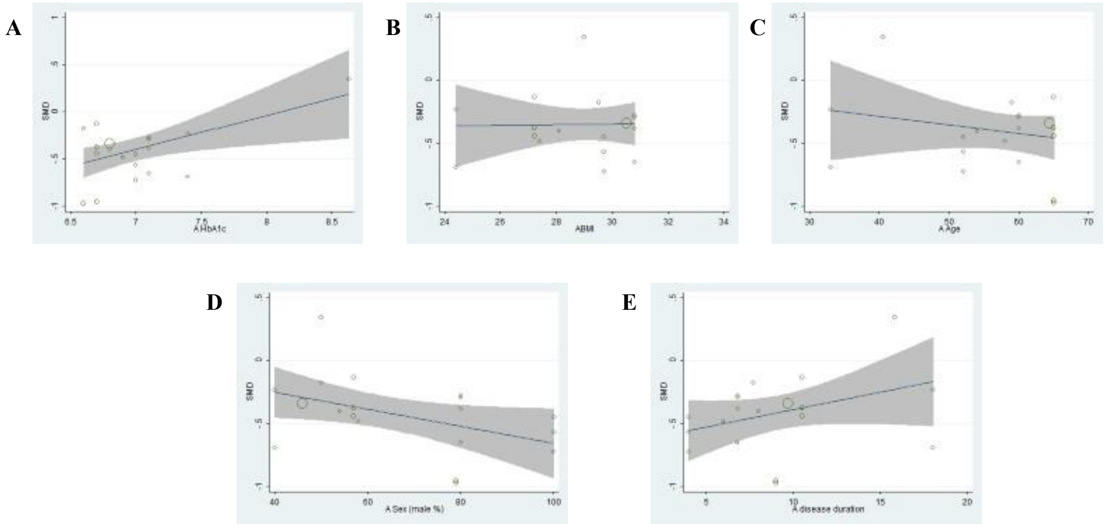


*HbA1c: Hemoglobin A1c; BMI: body mass index; TAR: time above range;*

Figure. S10 Associated factors with the change of TBR by meta-regression analysis. Associated factors: Baseline HbA1c (A), Baseline BMI (B), Baseline age (C), Baseline male percentage (D), and Disease duration (E)

A

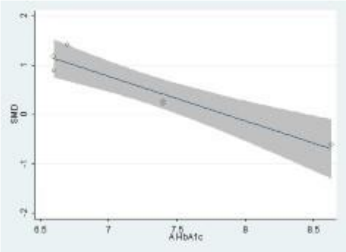

B

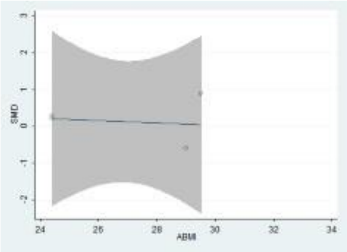

C

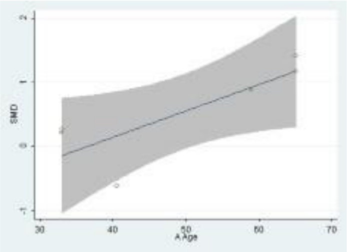

D

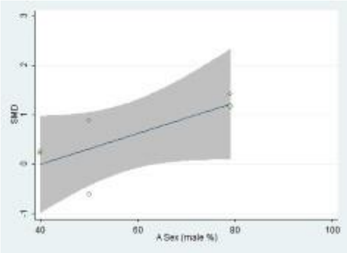

E

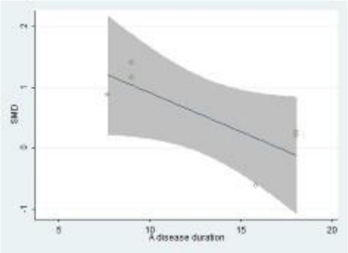

*HbA1c: Hemoglobin A1c; BMI: body mass index; TBR: time below range;*

## Reference


1. Dempsey PC, Blankenship JM, Larsen RN, Sacre JW, Sethi P, Straznicky NE, Cohen ND, Cerin E, Lambert GW, Owen N, et al. Interrupting prolonged sitting in type 2 diabetes: nocturnal persistence of improved glycaemic control. *Diabetologia* (2017) **60**:499–507. doi:10.1007/s00125-016-4169-z
2. Haxhi J, Leto G, di Palumbo AS, Sbriccoli P, Guidetti L, Fantini C, Buzzetti R, Caporossi D, Di Luigi L, Sacchetti M. Exercise at lunchtime: effect on glycemic control and oxidative stress in middle-aged men with type 2 diabetes. *Eur J Appl Physiol* (2016) **116**:573–582. doi:10.1007/s00421-015-3317-3
3. Rees JL, Chang CR, François ME, Marcotte-Chénard A, Fontvieille A, Klaprat ND, Dyck RA, Funk DR, Snydmiller G, Bastell K, et al. Minimal effect of walking before dinner on glycemic responses in type 2 diabetes: outcomes from the multi-site E-PAraDiGM study. *Acta Diabetol* (2019) **56**:755–765. doi:10.1007/s00592-019-01358-x
4. Li Z, Hu Y, Yan R, Li H, Zhang D, Li F, Su X, Ma J. Twenty Minute Moderate-Intensity Post-Dinner Exercise Reduces the Postprandial Glucose Response in Chinese Patients with Type 2 Diabetes. *Med Sci Monit* (2018) **24**:7170–7177. doi:10.12659/MSM.910827
5. Metcalfe RS, Fitzpatrick B, Fitzpatrick S, McDermott G, Brick N, McClean C, Davison GW. Extremely short duration interval exercise improves 24-h glycaemia in men with type 2 diabetes. *Eur J Appl Physiol* (2018) **118**:2551–2562. doi:10.1007/s00421-018-3980-2
6. Karstoft K, Clark MA, Jakobsen I, Müller IA, Pedersen BK, Solomon TPJ, Ried-Larsen M. The effects of 2 weeks of interval vs continuous walking training on glycaemic control and whole-body oxidative stress in individuals with type 2 diabetes: a controlled, randomised, crossover trial. *Diabetologia* (2017) **60**:508–517. doi:10.1007/s00125-016-4170-6
7. Myette-Côté É, Terada T, Boulé NG. The Effect of Exercise with or Without Metformin on Glucose Profiles in Type 2 Diabetes: A Pilot Study. *Can J Diabetes* (2016) **40**:173–177. doi:10.1016/j.jcjd.2015.08.015
8. Terada T, Wilson BJ, Myette-Côté E, Kuzik N, Bell GJ, McCargar LJ, Boulé NG. Targeting specific interstitial glycemic parameters with high-intensity interval exercise and fasted-state exercise in type 2 diabetes. *Metabolism* (2016) **65**:599–608. doi:10.1016/j.metabol.2016.01.003
9. Blankenship JM, Chipkin SR, Freedson PS, Staudenmayer J, Lyden K, Braun B. Managing free-living hyperglycemia with exercise or interrupted sitting in type 2 diabetes. *J Appl Physiol* (2019) **126**:616–625. doi:10.1152/japplphysiol.00389.2018
10. Munan M, Dyck RA, Houlder S, Yardley JE, Prado CM, Snydmiller G, Boulé NG. Does Exercise Timing Affect 24-Hour Glucose Concentrations in Adults With Type 2 Diabetes? A Follow Up to the Exercise-Physical Activity and Diabetes Glucose Monitoring Study. *Can J Diabetes* (2020) **44**:711-718.e1. doi:10.1016/j.jcjd.2020.05.012
11. Reddy R, Wittenberg A, Castle JR, El Youssef J, Winters-Stone K, Gillingham M, Jacobs PG. Effect of Aerobic and Resistance Exercise on Glycemic Control in Adults With Type 1 Diabetes. *Can J Diabetes* (2019) **43**:406-414.e1. doi:10.1016/j.jcjd.2018.08.193

12. Winding KM, Munch GW, Iepsen UW, Van Hall G, Pedersen BK, Mortensen SP. The effect on glycaemic control of low-volume high-intensity interval training versus endurance training in individuals with type 2 diabetes. *Diabetes, Obes Metab* (2018) **20**:1131–1139. doi:10.1111/dom.13198
13. Lee AS, Johnson NA, McGill MJ, Overland J, Luo C, Baker CJ, Martinez-Huenchullan S, Wong J, Flack JR, Twigg SM. Effect of High-Intensity Interval Training on Glycemic Control in Adults With Type 1 Diabetes and Overweight or Obesity: A Randomized Controlled Trial With Partial Crossover. *Diabetes Care* (2020) **43**:2281–2288. doi:10.2337/dc20-0342
